# Identification and genetic diversity of grapevine virus L in Greece

**DOI:** 10.1007/s00705-023-05756-z

**Published:** 2023-03-30

**Authors:** P. Panailidou, A. Galeou, D. Beris, P. Pappi, I. Theologidis, E. Tzagaki, L. Lotos, C. Varveri, N. I. Katis, V. I. Maliogka

**Affiliations:** 1 Laboratory of Plant Pathology, School of Agriculture, Faculty of Agriculture, Forestry and Natural Environment, Aristotle University of Thessaloniki, 54124 Thessaloniki, Greece; 2 Laboratory of Virology, Scientific Directorate of Phytopathology, Benaki Phytopathological Institute, 8 St. Delta str., 14561 Kifissia, Greece; 3 Laboratory of Plant Virology, Department of Viticulture, Vegetable Crops, Floriculture and Plant Protection, Hellenic Agricultural Organization DIMITRA (ELGO-DIMITRA), Institute of Olive Tree, Subtropical Crops and Viticulture, Kastorias 32A, Mesa Katsampas, 71307 Heraklion, Crete Greece; 4 Laboratory of Toxicological Control of Pesticides, Scientific Directorate of Pesticides’ Control and Phytopharmacy, Benaki Phytopathological Institute, 8 St. Delta str., 14561 Kifissia, Greece

## Abstract

In this study, grapevine virus L (GVL) was identified for the first time in Greece through the application of high-throughput sequencing of total RNA from grapevine samples. Further investigation of the prevalence of GVL in Greek vineyards by RT-PCR revealed its presence in 5.5% (31/560) of the tested samples, which originated from six viticultural areas of the country. Comparative sequence analysis based on the CP gene revealed a high degree of genetic variability among GVL isolates, while phylogenetic analysis grouped the Greek isolates in three of the five phylogroups formed, with most of them being classified in phylogroup I.

**Supplementary Information:**

The online version contains supplementary material available at 10.1007/s00705-023-05756-z.

Grapevine (Vitis *vinifera* L.) is cultivated worldwide and is known to be infected by at least 86 viruses from 17 families [1]. The genus *Vitivirus* of the family *Betaflexiviridae* (subfamily *Trivirinae*) includes ten viral species whose members infect grapevine. Grapevine virus A (GVA), grapevine virus B (GVB), grapevine virus D (GVD), grapevine virus E (GVE), grapevine virus F (GVF), grapevine virus G (GVG), grapevine virus H (GVH), grapevine virus I (GVI), grapevine virus J (GVJ), and grapevine virus L (GVL) are officially members of the genus according to the International Committee on Taxonomy of Viruses (ICTV) (https://talk.ictvonline.org/taxonomy/), while grapevine virus K (GVK), grapevine virus M (GVM), grapevine virus N (GVN), and grapevine virus O (GVO) have recently been identified and proposed to be classified in this genus [2–5]. Vitiviruses have a +ssRNA genome that is 7,300 to 7,600 nucleotides (nt) long and is encapsidated in non-enveloped flexuous filamentous virions [6, 7]. The genome is organized into five open reading frames (ORFs), flanked by a 5’-end methylated cap and a 3’-end poly-A tail. The genome encodes a protein responsible for viral replication (ORF1), an 18- to 22-kDa protein with unknown function (ORF2), a movement protein (MP) (ORF3), a coat protein (CP) (ORF4), and a nucleic-acid-binding protein (NABP) (ORF5) [7]. GVA, GVB, and GVD are involved in the rugose wood (RW) disease complex [8, 9], and GVA, GVB, GVE, GVG, and GVH are known to be transmitted by mealybugs (Hemiptera: Pseudococcidae) and soft scale insects (Hemiptera: Coccidae) [9–12].

GVL is a newly identified member of the genus *Vitivirus* whose genome sequence was first identified in publicly available RNAseq libraries of grapevine samples from China, Croatia, USA (a Canadian grapevine sample), and New Zealand [3]. Since then, additional GVL isolates from grapevine samples from the USA (California and Texas) [13, 14], Tunisia [15], Turkey [16], South Africa [17], Korea [18], and France [19] have been characterized. The GVL genome is 7,607 nt long and has a genome organization that is typical of members of the genus *Vitivirus*. ORF2 encodes a 22-kDa protein and overlaps at the tetranucleotide AUGA with ORF1, a feature that has also been observed in the genome sequence of GVJ [3]. To date, no information is available about the transmission of GVL and its association with any grapevine disease.

In the past few years, several vitiviruses, namely GVA, GVB, GVE, GVF, GVI, and GVH, have been reported in Greek vineyards [20–25]. In this study, GVL was initially identified using high-throughput sequencing (HTS), and a broader survey was then conducted to determine the diversity and phylogenetic relationships among populations.

In 2020, HTS was performed on two individual and three composite grapevine samples that were collected randomly from different viticultural areas of Greece. The individual samples originated from the national grapevine germplasm collection of the Viticulture Department of Athens, the Institute of Olive Tree, Subtropical Crops and Viticulture (ΙΟSV) (ELGO-DEMETER) in Lykovrisi Attica (sample AG-1, cv. Agiorgitiko) and from the grapevine germplasm collection of the Aristotle University of Thessaloniki (A.U.TH.) (sample AUTH69, cv. Bekari). Two of the three composite samples of cv. Assyrtiko were collected from commercial vineyards in central Macedonia (samples GeA and XA), and the last one consisted of three different cultivars collected from Crete (sample PKs1, cv. Alatsatiano, cv. Vidiano, and cv. Assyrtiko).

For HTS analysis, total RNA was extracted from leaf, petiole, or phloem scraping tissue (fresh or freeze-dried). More specifically, 0.2 g of tissue was used for the individual samples. For composite sample PKs1, phloem scrapings (0.1 g) from each vine were used, whereas for the composite samples GeA and XA, 0.1 g of freeze-dried tissue from each vine was used. The freeze-dried tissue mix was ground into powder using a pestle and mortar, the powder was vortexed, and 0.05 g of this material was used for RNA extraction. Samples GeA, XA, and PKs1 were comprised of nine, eleven, and three vines, respectively.

For total RNA extraction, the protocol developed by Ruiz-García et al. [26] was applied, with a few modifications (Supplementary Text). Approximately 25 μL of each sample were added to an RNAstable (Biomatrica Inc.) or GenTegraRNA (GenTegra® LLC.) tube, and the sample was dried using a vacuum desiccator. The tubes were shipped at room temperature to Macrogen Inc. (Seoul, S. Korea) for rRNA depletion, library construction, and high-throughput sequencing (HTS) on a NovaSeq6000 (Illumina, Inc.) platform. The selected yield for the samples was ~50 million 100-nucleotide (nt)-long paired-end (PE) reads.

For the AG1 sample, total RNA was extracted from 0.1 g of freeze-dried tissue using the CTAB-based protocol described by Gambino et al. [27]. Ribosomal RNA (rRNA) was removed using a RiboMinus™ Plant Kit for RNA-Seq (Thermo Fisher Scientific), and the resulting ribo-depleted RNA was sent to the Greek Genome Center (Biomedical Research Foundation Academy of Athens, BRFAA) for HTS analysis on a NovaSeq6000 (Illumina, Inc.) platform, generating ~25 million 100-nucleotide single-end (SE) reads.

The quality of the HTS reads was assessed using FastQC [28], and the reads were trimmed for quality and deduplicated using PRINSEQ-lite [29]. Reads corresponding to host sequences were removed using Geneious Prime (Dotmatics), *de novo* assembly was performed using SPAdes (v. 3.14.1) [30], and the contigs were subjected to a similarity search against the nt database using BLASTn locally.

For confirmation of the presence of GVL in single and composite samples, total RNA was extracted from leaf, petiole, or phloem tissue scrapings of each sample (depending on the sampling season), using the extraction method described above and the one-step reverse transcription polymerase chain reaction (RT-PCR) procedure described by Ilbagi et al. [16], using a set of primers (GVL_F_6750/GVL_R_6938, Table 1) that amplify a 189-bp-long fragment of the GVL *CP* gene (ORF4). One GVL isolate that was detected using the above reaction in all of the composite samples analyzed by HTS (GB15 from pool GeA, X10 from pool XA, and Ks14 from PKs1) and in the two isolates retrieved from the individual samples analyzed by HTS (AG-1 and AUTH69) were selected for confirmation of a larger part of the genome sequence by Sanger sequencing. For this purpose, two new reactions were developed in order to amplify either the complete (670 nt long) *CP* gene of GVL, using the newly designed primers GVL_F_6495/GVL_R_7167 (Table 1) in a two-step RT-PCR assay (Supplementary Text) (samples GB15, X10, AG-1 and AUTH69), or a 883-nt fragment of the GVL *MP* gene and the 5’-terminal portion of the *CP* gene, using the newly designed primers GVL UP/28V and GVL UP NEST/GVL DO NEST (Table 1) in a RT-PCR assay and a subsequent nested PCR assay, respectively (Supplementary Text) (sample Ks14). In all cases, the final reaction volume was 100 μl (5 tubes of 20 μl), and DNA was purified using a Monarch® PCR & DNA Cleanup Kit (New England Biolabs Inc.) according to the manufacturer’s instructions. Then, purified DNA was sequenced in both directions by the Sanger method by either GENEWIZ (Leipzig, Germany) or Eurofins Genomics (Ebersberg, Germany), and the sequence of each Greek isolate was compared to sequences in the GenBank database, using the BLASTn algorithm, as well as to the corresponding nucleotide sequences obtained by HTS analysis.

**Table 1:** Primers used in RT-PCR assays in this study

Purpose of assay	Name of primer	Sequence (5'-3')	ORF - gene target	Αmplicon length (bp)	Reference
Detection	GVL_F_6750	AGC DGG TGA KCC TCT TAA T	ORF4 - coat protein	189	Ilbağı et al. [16]
	GVL_R_6938	G TCA TCT TCC TAG CYA GRC			
Sequencing	GVL_F_ 6495	GTGCGAAGRGCAATARAC	ORF4 - coat protein	670	This study
	GVL_R_7167	TAGACTCACCCATATAMYTMTC			
	GVL_CP-Var-Up	GATGATGCACTTATGTCKGACG	ORF4 - coat protein	712	This study
	GVL_CP-Var-Do	CYCTACGYTTAYTAGCACTYCTAG			
	GVL UP	CKTTYAAGGTGAAGGGGAG	ORF3-3'UTR - movement protein - coat protein - RNA binding protein	1820	This study
	28V	GGGGATCCGCGGTTTTTTTTTTTTTTTT			
	GVL UP NEST	GGGAGCAARAATGGWCTSAG	ORF3-ORF4 - movement protein & coat protein	883	This study
	GVL DO NEST	GWARCAGGGCACACTGG			
	Oligo(dT) 18-mer	TTT TTT TTT TTT TTT TTT			

In order to investigate the presence of GVL in Greek vineyards and germplasm collections, a total of 560 grapevine samples were collected from grapevine germplasm collections and commercial vineyards in 13 regions of Greece from 2015 to 2020 (Table 2). More specifically, plant material from 111 vines originated from the national grapevine germplasm collection of ΙΟSV (ELGO-DEMETER) in Lykovrisi, Attica. In addition, 32 grapevine samples were acquired from the grapevine germplasm collection of A.U.TH., and 80 samples were collected from the grapevine germplasm collection of IOSV (ELGO-DEMETER) in Heraklion, while the rest of the samples came from commercial vineyards. Most of the collected samples (433) came from grafted Greek varieties, and 127 were collected from self-rooted Greek and foreign varieties (Table 2). All samples were tested for the presence of GVL using the one-step RT-PCR assay (Supplementary Text) described by Ilbağı et al. [16] (Table 1).

**Table 2:** Grapevine material collected and tested for the presence of grapevine virus L (GVL)

Sampling area	Υear of collection	Cultivar	Number of vineyards or grapevine collections	Number of cultivars	Plant tissue	GVL positive/number of tested samples	
						Grafted	Self-rooted
Amyntaio	2015	Foreign	1 - V	4	Leaves & stems	0/38	-
Attica	2016 & 2020	Greek	1 - C	51	Phloem scrapings	14/111	-
Heraklion	2019	Greek	3 - V & 1 - C	35	Leaves	3/80	2/80
Kavala	2019	Foreign	1 - V	1	Leaves	0/10	-
Kilkis	2019	Greek	3 - V	3	Leaves & stems	2/19	-
Lasithi	2019	Greek	7 - V	15	Phloem scrapings	-	0/26
Mantineia	2020	Greek	8 - V	1	Leaves, phloem scrapings	0/11	-
Naousa	2017	Foreign & Greek	8 - V	8	Leaves	0/29	-
Nemea	2017 & 2020	Greek	16 - V	3	Leaves, phloem scrapings	2/41	0/15
Thessaloniki	2019-2020	Greek & Foreign	3 - V & 1 - C	21	Leaves, stems & phloem scrapings	5/49	1/6
Thira	2019	Greek	1 - V	1	Leaves, phloem scrapings	-	0/22
Tirnavos	2020	Greek	1 - V	1	Leaves	2/6	-
Chalkidiki	2017	Foreign	1 - V	1	Phloem scrapings	0/17	-
**Total (%)**						**28/411 (6.8%)**	**3/149 (2%)**
						**31/560**	
						**(5.5%)**	

*V, commercial vineyard; C, collection

To investigate the genetic variability of the GVL *CP* gene, 10 isolates were selected for Sanger sequencing, while the sequences of another three isolates obtained from the HTS analysis (AG-1, AUTH69/1 and PKs1-8) were also included (Supplementary

Table S1). The complete *CP* gene of GVL (primers GVL_F_6495/GVL_R_7167, Table 1) was amplified from seven samples collected from Attica, Kilkis, and Thessaloniki from 2016 to 2020 (Supplementary Table S1). A new two-step RT-PCR assay (Supplementary Text) was also designed to amplify the complete *CP* gene of GVL (712-nt fragment, primers GVL_CP-Var-Up/GVL_CP-Var-Do, Table 1) from three isolates from Tyrnavos and Thessaloniki (Supplementary Table S1). For all selected isolates, PCR amplicons were purified, and Sanger sequencing was performed as described above.

GVL sequences obtained by PCR and HTS were analyzed using MEGA Χ software [31], and the sequences of the Greek isolates (Supplementary Table S1) and those of other isolates obtained from the GenBank database (https://www.ncbi.nlm.nih.gov/) were compared using Geneious Prime software (https://www.geneious.com/prime/) after alignment using MAFFT. A phylogenetic tree was constructed based on the *CP* gene nucleotide sequences of the Greek isolates determined in this study and those of other isolates obtained from GenBank. The alignment, the selection of the substitution model, and the construction of the phylogenetic tree were carried out using the MEGA X bioinformatics suite [31]. The best nucleotide substitution model was found using the option Find Best DNA/Protein Models (ML). The maximum-likelihood method was selected for constructing the phylogenetic tree, using the model K2+G+I, while a non-parametric bootstrap analysis of 1,000 repetitions was performed for the evaluation of the reliability of the phylogenetic hypothesis.

Analysis of HTS results revealed the presence of GVL in Greek vineyards and germplasm collections. The HTS runs yielded 25-60 million reads for each sample, with *de novo* assembly producing 44,311, 614, 468, 2,179, and 1,779 contigs for samples AG-1, AUTH69, PKs1, XA, and GeA, respectively. BLASTn results revealed the presence of nearly full-genome-length contigs of GVL for samples AG-1, AUTH69, Pks1, and XA, with more than 91% nucleotide sequence identity to the GVL-VL isolate (MH681991.1) from Croatia. In GeA, the contigs were of variable size (246-5,121 nt) with 87.6-98.8% nucleotide sequence identity to the GVL-VL isolate. Using a one-step RT-PCR reaction [14], GVL was detected in the individual samples AUTH69 and AG-1 and in one of the three samples comprising the composite sample PKs1 (Ks14), in two of the 11 samples comprising the composite sample XA (X10 and X11), and in three of the nine samples comprising the composite sample GeA (GB15, GB20, and GB21). A 670-nt fragment of the *CP* gene was amplified using samples AUTH69, AG-1, X10, and GB15, and an 883-nt fragment of the *MP* and *CP* genes was also amplified from sample Ks14. All of the sequences obtained from the amplicons were identical to the corresponding contigs obtained by HTS analysis. BLASTn analysis revealed that the sequenced isolates had a high degree of similarity to GVL isolates with sequences in the GenBank database. Specifically, isolates PKs1-8 and GB15 had 95.41% and 98.73% nt sequence identity, respectively, to GVL isolate VL, isolates AG-1 and AUTH69/1 showed 97.32% and 96.03% sequence identity, respectively, to isolate Marsaoui (ΜΤ319082.1, Tunisia), and isolate X10 showed 96.98% nt sequence identity to isolate Red Blotch (ΜΤ319081.1, Tunisia).

Further analysis of the prevalence of GVL in Greek vineyards revealed its presence in 5.5% (31/560) of the tested samples (Table 2). The virus was mostly detected in samples collected from grafted vines (6.8%, 28/411) and in a small number of samples from self-rooted vines (2%, 3/149). In addition, GVL was mainly identified in Greek grapevine varieties and in only one foreign cultivar (Calmeria). As for its geographic distribution, GVL was found in six different regions of Greece (Table 2). Most of the virus isolates originated from the vineyard of the grapevine germplasm collection of ΙΟSV (ELGO-DEMETER) in Attica (14/111), while GVL was also detected in six samples from Thessaloniki (three from a commercial vineyard and three from the vineyard of A.U.TH.), five samples from Heraklion (three from a commercial vineyard and two from the collection), six samples from commercial vineyards in Kilkis, Naousa, and Tyrnavos (two samples per vineyard).

The nucleotide sequence of the *CP* gene of 11 GVL genotypes was determined by Sanger sequencing. In the sample GB21, two divergent sequences were identified. Comparative analysis of the *CP* sequences, including the ones obtained from AG-1, AUTH69, and Ks14 by HTS, revealed 82–99.5 % nt sequence identity and 88.5–100% amino acid (aa) sequence identity among the Greek isolates (Supplementary Tables S2 and S3). “GVL-3” was found to be the most divergent of the isolates. The *CP* nt and aa sequence identity between Greek isolates and those identified in other countries ranged from 80.83 to 99% and from 87 to 100%, respectively (Supplementary Tables S2 and S3).

The *CP*-based phylogenetic tree grouped the isolates into five distinct groups (Fig. 1), four of which were in agreement with those based on ORF1 reported by Read et al. [17] and named accordingly. Most of the Greek isolates of GVL clustered together with isolates from South Africa, Croatia, and Tunisia in phylogenetic group I, while phylogroup IV was formed by isolate "5.G4-1" together with isolates from Canada, the USA, France, China, and South Africa (Fig. 1). The isolates “GVL-3” from Greece and “SB” (MH686191.1) from New Zealand were classified in group V, while phylogroups II and III included only isolates from the USA and South Africa, respectively (Fig. 1).

**Fig. 1:**
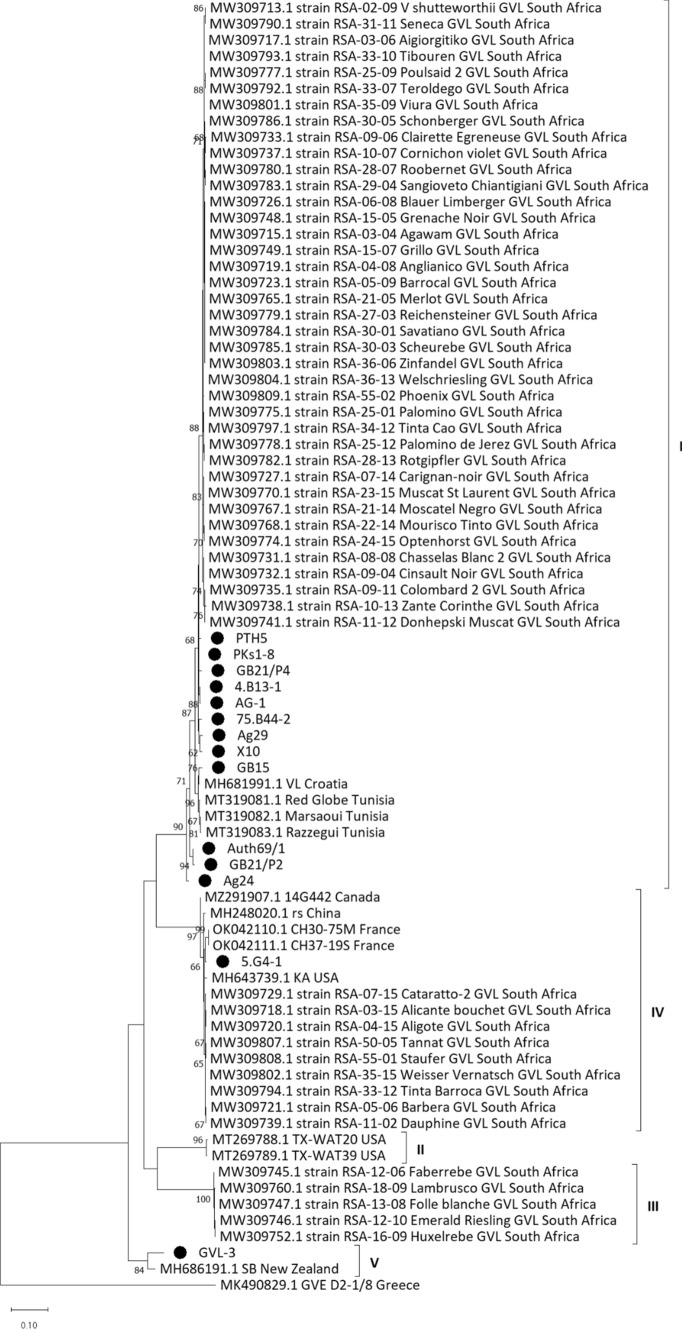
Maximum-likelihood phylogenetic tree based on complete nucleotide sequences of the coat protein gene of grapevine virus L (GVL). Greek isolate sequences of GVL (indicated by black circles) and other GVL sequences from different countries (referred to by their GenBank accession number, isolate name, and origin) were used for this analysis. The percentage of 1000 repetitions of bootstrap analysis that supports grouping at each node is indicated. The scale bar represents the number of nucleotide substitutions per position, while the length of the branches is proportional to the genetic distances that were calculated. Isolate MK490829.1 of GVE was used as an outgroup. A group of 24 South African isolates that share 100% nucleotide sequence identity are represented by isolate MW309717.1, a second group of 14 South African isolates that share 100% nucleotide sequence identity are represented by isolate MW309747.1, and a third group of five South African isolates that share 100% nucleotide sequence identity are represented by isolate MW309770.1.

In this study, GVL was identified for the first time in Greek vineyards, thus further expanding our knowledge about the geographic distribution of this virus. Greece is the tenth country in which GVL has been detected, after Canada, Croatia, New Zealand, the USA [3, 13, 14], Tunisia [15], Turkey [16], South Africa [17], Korea [18], and France [19]. In addition, the identification of GVL has increased the number of vitiviruses that are known to be endemic in Greek vineyards to seven [20–25].

GVL was detected in only 5.5% of the samples tested in our study, mainly in Greek grapevine varieties, while it seems to be present in several geographical regions of central Greece, Macedonia, and Crete. The prevalence of GVL in Greek vineyards is similar to that of the newly reported GVE and GVI, based on initial data from small-scale surveys [23, 24], whereas GVA and GVB have been detected at a higher frequency in Greek vineyards (38.5% and 20.1%, respectively) and exhibit a wide distribution in the Greek territory [32].

Although GVL exhibits a worldwide distribution, its prevalence is usually low, as observed in previous studies [3, 13, 16]. Higher frequencies have been recorded in a few studies in which the samples were collected from the same vineyard/germplasm collection or originated from the same plant material [17, 18], suggesting that the use of infected plant material or the presence of a vector might increase the incidence of GVL. In our study, the virus exhibited a higher frequency (12.6%) within the national germplasm collection, indicating that, at least in this region, it has been present for an extended period of time.

The coexistence of several GVL variants in the same population observed in this work could be attributed to the grafting of infected material or secondary infections through a putative virus vector. This phenomenon seems to be characteristic of grapevine viruses of the genus *Vitivirus*, as it has been reported to occur in the cases of GVA and GVB [33, 34] as well as GVE and GVF in Greek grapevine samples (Panailidou et al., unpublished data).

Genetic diversity in the *CP* gene was observed among Greek isolates and between Greek and foreign isolates, at both the nt and aa level, but some isolates showed a high percentage of similarity (Supplementary Tables S2 and S3). This is reflected in the phylogenetic tree that was constructed using the same genomic region of GVL, as the five phylogroups were separated by large genetic distances, with small genetic distances, separating the isolates within each phylogroup (Fig. 1). The high genetic variability among GVL isolates has also been noted recently by Debat et al. [3] and Alabi et al. [14], and a high level of sequence similarity within the *CP* gene has been described by Debat et al. [3], Diaz-Lara et al. [13], and Alabi et al. [14]. The phylogenetic groups identified in the present study are in accordance with previous studies, with phylogroups I, II, III, and IV reported previously by Read et al. [17], phylogroup I by Ben Amar et al. [15], and phylogroup II by Alabi et al. [14] (Fig. 1). Although most of the Greek isolates were classified as belonging to phylogroup I, together with the majority of other GVL sequences, two isolates grouped with members of clusters IV and V, suggesting that there have been multiple introductions of GVL in Greek vineyards through infected plant material.

In summary, GVL is a new but highly divergent virus of grapevine that is present in Greece and several other countries. Given its variability, special attention should be paid to the application of reliable molecular methods for its accurate identification. In addition, the pathogenicity of GVL to grapevine remains unknown. The coexistence of GVL with other known grapevine-infecting viruses, as documented in other studies [14, 17] and also observed here (data not shown), makes it difficult to assess its pathogenicity. Future research should focus on characterization of the biological properties of GVL, including its putative vector transmission, as well as on its interaction with other grapevine viruses.

## Supplementary Information

Below is the link to the electronic supplementary material.

**Supplementary Table S1** Isolates of grapevine virus L (GVL) subjected to HTS analysis and Sanger sequencing and their respective GenBank accession numbers. **Supplementary Table S2** Percentage of nucleotide sequence identity between Greek and foreign isolates of GVL. The values refer to the complete CP sequences of Greek GVL isolates, determined by Sanger sequencing and HTS (in bold letters) and those of foreign isolates obtained from the GenBank database (indicated by their accession numbers). The sequences were aligned using MAFFT and analyzed using Geneious Prime software. **Supplementary Table S3** Percentage of amino acid sequence identity between Greek and foreign isolates of GVL. The values refer to partial replicase sequences of Greek GVL isolates, which were determined by Sanger sequencing and HTS (in bold letters) and those of foreign isolates obtained from the GenBank database (indicated by their accession numbers). The sequences were aligned using MAFFT and analyzed using Geneious Prime software. (XLSX 120 KB)

Detailed description of the RT-PCR assays used in this study and the modified total RNA extraction protocol of Ruiz-García et al. [26] (DOCX 15 KB)

## Data Availability

The nucleotide sequences reported here have been deposited in the GenBank database under the accession numbers OP893976, OP893977, OP893978, OP893979, OP893980, OP893981, OP893982, OP893983, OP893984, OP893985, OP893986, OP893987, OP893988, and OP893989.
