# Supplementary material for: Identification and genetic diversity of grapevine virus L in Greece
Source: Arch Virol. 2023 Mar 30;168(4):127. doi: 10.1007/s00705-023-05756-z (PMC10063476; doi:10.1007/s00705-023-05756-z)
Supplement: Supplementary file 2 — Detailed description of the RT-PCR assays used in this study and the modified total RNA extraction protocol of Ruiz-García et al. [26] (DOCX 15 KB) [file 705_2023_5756_MOESM2_ESM.docx]

**Supplementary Text**

Modifications of total RNA extraction protocol developed by Ruiz-García et al [26]

Briefly, after step 11 (§3.2) 0.8 volumes of 2-propanol (PanReac-AppliChem) were added to the supernatant and 2 ml of the mixture were transferred immediately to a Monarch® RNA Purification Column (New England Biolabs Inc.). The column was centrifuged at 14,000 x g for 2 min and then washed with 700 μl of Wash-Solution 2 (Merck KGaA). Afterwards a DNA digestion step was performed using the On-Column DNase I Digestion Set (Merck KGaA) according to the manufacturer’s specification, followed by two columns wash using 700 μl of Wash-Solution 2. The RNA was eluted by adding 60 μl RNase-Free water and centrifuging the column at 12,000 x g for 2 min. Moreover, when freeze-dried tissue was used for the extraction the plant tissue:CTAB buffer ratio used was 1:100 (w/v).

One-step RT-PCR, primers GVL_F_6750/GVL_R_6938

The reaction took place by mixing 5U of MMLV Reverse Transcriptase (GeneON BioScience), 2.5 U Taq DNA Polymerase (GenScript), 1 μM of primers GVL_F_6750 and GVL_R_6938 (Table 1), 0.25 mM of each dNTP, 1X of Standard Taq Reaction Buffer (10 mM Tris-HCl (pH 8.3), 50 mM KCl, 1.5 mM MgCl_2_) (New England Biolabs Inc.), 3 μl of extracted RNA and nuclease free water to a final volume of 25 μl, following the thermocycle profile of 42^o^C for 50 min, 95°C for 3 min, 40 cycles of 95°C for 20 s, 56°C for 10 s, 52°C for 10 s, 72°C for 10 s, and a final step of 72°C for 2 min.

Two-step RT-PCR, primers GVL_F_6495/GVL_R_7167

Three (3) μl of total RNA was mixed with 40 U M‐MLV (GeneON), 1 μM of Oligo(dT) 18-mer primer (Table 1), 0.25 mM of each dNTP, 1X standard buffer [50 mM Tris-HCl (pH 8.3), 75 mM KCl, 3 mM MgCl_2_] and nuclease free water to a final volume of 20 μl in RT reaction, with a thermocycle profile of 42°C for 60 min and 70°C for 15 min. After RT, a PCR reaction was carried out in a final volume of 20 μl, which contained 1.5 U Taq DNA Polymerase (GenScript), 1 μM of primers GVL_F_6495 and GVL_R_7167 (Table 1), 0.2 mM of each dNTP, 1X of Standard Taq Reaction Buffer (10 mM Tris-HCl (pH 8.3), 50 mM KCl, 1.5 mM MgCl_2_) (New England Biolabs Inc.), 2 μl of the product of RT reaction and nuclease free water to a final volume, following the thermocycle profile of 95°C for 3 min, 40 cycles of 95°C for 30 s, 56°C for 15 s, 52°C for 15 s, 72°C for 30 s, and a final step of 72°C for 3 min.

One-step RT-PCR assay followed by a nested PCR, primers GVL UP/28V and GVL UP NEST/GVL DO NEST

A RT-PCR assay was held by mixing 5 U of MMLV Reverse Transcriptase (GeneON BioScience), 2.5 U Taq DNA Polymerase (GenScript), 1 μM of primers GVL UP and 28V (Table 1), 0.25 mM of each dNTP, 1X of Standard Taq Reaction Buffer (10 mM Tris-HCl (pH 8.3), 50 mM KCl, 1.5 mM MgCl_2_) (New England Biolabs Inc.), 3 μl of extracted RNA and nuclease free water to a final volume of 25 μl, following the thermocycle profile of 42^o^C for 60 min, 95°C for 3 min, 40 cycles of 95°C for 30 s, 52°C for 30 s, 72°C for 1 min, and a final step of 72°C for 5 min, followed by a nested PCR in which 1.5 U Taq DNA Polymerase (GenScript) mixed with 1 μM of primers GVL UP NEST/GVL DO (Table 1), 0.2 mM of each dNTP, 1X of Standard Taq Reaction Buffer (10 mM Tris-HCl (pH 8.3), 50 mM KCl, 1.5 mM MgCl_2_) (New England Biolabs Inc.), 1μl of the product of RT-PCR reaction and nuclease free water to a final volume of 20 μl, following the thermocycle profile of 95^o^C for 3 min, 40 cycles of 95^o^C for 30 s, 56^o^C for 30 s, 72^o^C for 50 s, and a final step of 72^o^C for 5 min.

Two-step RT-PCR, primers GVL_CP-Var-Up/GVL_CP-Var-Do

In RT reaction 3 μl of extracted RNA was mixed with 40 U M‐MLV (GeneON), 1 μM of OligodT (18mer) primer (Table 1), 0.25 mM of each dNTP, 1X standard buffer [50 mM Tris-HCl (pH 8.3), 75 mM KCl, 3 mM MgCl_2_] and nuclease free water to a final volume of 20 μl, followed by the thermocycle program: 42^ο^C for 60 min and 70^o^C for 15 min. For the PCR reaction 2 μl of the previous reaction were added in a reaction mixture which contained 1.5 U Taq DNA Polymerase (GenScript), 0.8 μM of primer GVL_CP-Var-Up and 1 μM of primer GVL_CP-Var-Do (Table 1), 0.2 mM of each dNTP, 1X of Standard Taq Reaction Buffer (10 mM Tris-HCl (pH 8.3), 50 mM KCl, 1.5 mM MgCl_2_) (New England Biolabs Inc.), and nuclease free water to a final volume of 25 μl. PCR was performed using the following parameters: 95^o^C for 3 min, 40 cycles of 95^o^C for 30 s, 61^o^C for 10 s, 59^o^C for 10 s, 55^o^C for 10 s, 72^o^C for 35 s, and a final step of 72^o^C for 3 min.
